# Size Structure of Marine Soft-Bottom Macrobenthic Communities across Natural Habitat Gradients: Implications for Productivity and Ecosystem Function

**DOI:** 10.1371/journal.pone.0040071

**Published:** 2012-07-20

**Authors:** Tara A. Macdonald, Brenda J. Burd, Albert van Roodselaar

**Affiliations:** 1 Institute of Ocean Sciences, Department of Fisheries and Oceans, Sidney, British Columbia, Canada; 2 Biologica Environmental Services, Victoria, British Columbia, Canada; 3 Ecostat Research Limited, North Saanich, British Columbia, Canada; 4 Metro Vancouver, Burnaby, British Columbia, Canada; Argonne National Laboratory, United States of America

## Abstract

Size distributions of biotic assemblages are important modifiers of productivity and function in marine sediments. We investigated the distribution of proportional organic biomass among logarithmic size classes (2^−6^J to 2^16^J) in the soft-bottom macrofaunal communities of the Strait of Georgia, Salish Sea on the west coast of Canada. The study examines how size structure is influenced by 3 fundamental habitat descriptors: depth, sediment percent fines, and organic flux (modified by quality). These habitat variables are uncorrelated in this hydrographically diverse area, thus we examine their effects in combination and separately. Cluster analyses and cumulative biomass size spectra reveal clear and significant responses to each separate habitat variable. When combined, habitat factors result in three distinct assemblages: (1) communities with a high proportion of biomass in small organisms, typical of shallow areas (<10 m) with coarse sediments (<10% fines) and low accumulation of organic material (<3.0 gC/m^2^/yr/δ^15^N); (2) communities with high proportion of biomass in the largest organisms found in the Strait, typical of deep, fine sediments with high modified organic flux (>3 g C/m^2^/yr/δ^15^N) from the Fraser River; and (3) communities with biomass dominated by moderately large organisms, but lacking the smallest and largest size classes, typical of deep, fine sediments experiencing low modified organic flux (<3.0 gC/m^2^/yr/δ^15^N). The remaining assemblages had intermediate habitat types and size structures. Sediment percent fines and flux appear to elicit threshold responses in size structure, whereas depth has the most linear influence on community size structure. The ecological implications of size structure in the Strait of Georgia relative to environmental conditions, secondary production and sediment bioturbation are discussed.

## Introduction

In marine soft-bottom habitats, benthic macrofauna may range in size over several orders of magnitude [1]–[3]. The distribution of biomass across size classes, or the biomass size spectrum, is considered an emergent ecological property of these communities [2], [4] because it can influence system dynamics, productivity, function, and stability across widely different ecosystems (e.g., [5]–[9]).

The high prevalence of small organisms in a benthic community may be indicative of habitat instability [2] and results in high production relative to total biomass (given the high turnover rates of member organisms [7]. In contrast, communities dominated by large organisms, although potentially having higher total biomass, may have lower relative production due to long life-spans and slow turnover rates of organisms. Large animals may also facilitate productivity, having substantial impacts on the structure, aeration and geochemistry of sediments through the formation, maintenance and ventilation of burrows (Examples include spantagoid urchins[10]–[12]; and Arenicolid polychaetes [13]). These large animals may be considered ecosystem engineers, as they play a vital role in the maintenance of regional bio-diversity [14], [15] by adding heterogeneity to the structure of benthic habitats [16].

At this point in our history, there is a growing imperative to understand baseline biological conditions in our oceans. Without this context, we cannot predict or mitigate the effects of anthropogenic stressors or changes in climate. The size structure of marine macrobenthic communities is affected by anthropogenic stressors such as organic enrichment [17]–[21] and trawling [22]–[24]. Recent evidence from freshwater systems suggests climate warming could also cause significant shifts in benthic community size structure [25]. Such shifts in size structure could have significant impacts on marine ecosystems, affecting sediment production, geochemistry, and the amount of food available to predators at higher trophic levels [26]. In order to understand the potential effects of these impacts, we must first establish how size structure varies across natural gradients, and what variations in size structure reveal about the functioning of soft-bottom habitats.

Previous authors have found macrobenthic size structure is influenced by depth (e.g., [4], [9], [27]), which is presumably associated with food availability and habitat stability. The role of sediment type, however, is less clear, since it co-varies with other habitat factors that may influence size spectra [28]. It remains debatable whether a mechanistic link between size structure and sediment granulometry can be isolated when other environmental factors are considered [4], [29]. This study examines the relative importance of three key environmental factors (depth, granulometry and food availability) on marine macrobenthic size structure across a large coastal region.

The Strait of Georgia, British Columbia is a hydrographically diverse coastal sea on the west coast of Canada, incorporating a wide range of sediment production and biomass conditions, including habitats exposed to high organic and inorganic loading from the Fraser River in the southern Strait, and very low organic flux in the northern Strait [30], [31].The purpose of this paper is to determine if and how macrobenthic size structure varies significantly across gradients in depth, sediment percent fines, and organic flux and quality over this broad geographic region. This is accomplished using an existing extensive database of macrobenthic and related sediment data spanning depth ranges of 0–678 m, substrate with 0.1–100% fines content, and organic flux to sediments of 0.1–13 gC/m^2^/yr [30], [31]. We focus on regions not directly under the influence of anthropogenic stressors (‘background’ regions; [30]–[32]) in order to establish baseline conditions of macrobenthic size structure for the Strait of Georgia.

## Materials and Methods

### Description of Database & Size Categorization

**Figure 1:**
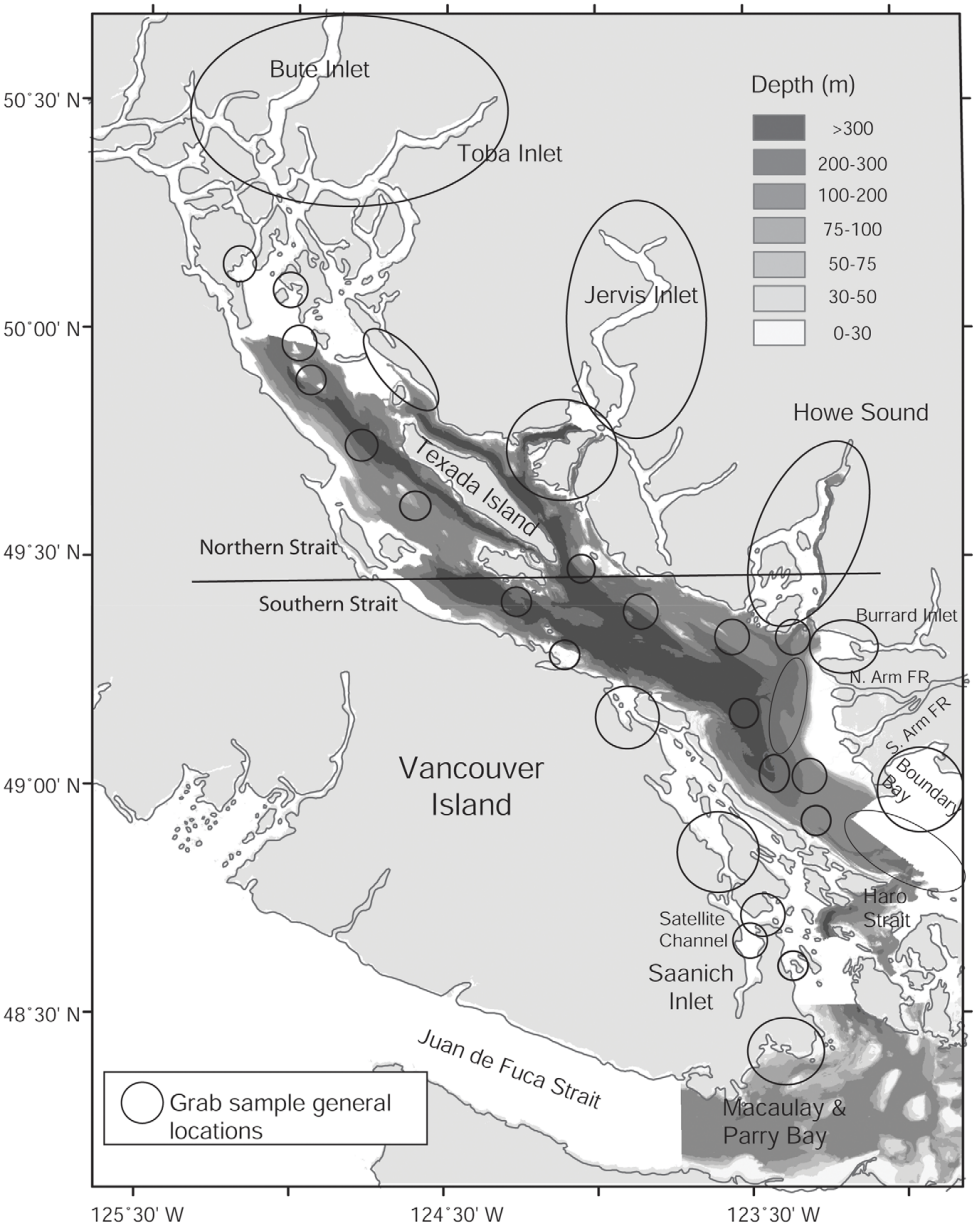
Map of general study locations of macrobenthic grab sampling surveys compiled in the Strait of Georgia database (coastal British Columbia, Canada). Circles indicate locations of grab samples. Line indicates demarcation between Northern and Southern Strait of Georgia.

The BC coastal database is maintained and updated at the Institute of Ocean Sciences, Sidney, British Columbia (Fisheries and Oceans Canada; contact Brenda.Burd@dfo-mpo.gc.ca), and contains macrofaunal and related habitat data as described by Burd et al. [30], [31]. This study focuses on a subset of samples included in this database from areas in the Strait of Georgia not directly under the influence of localized anthropogenic inputs. These background biological and associated sediment data were from grab samples collected primarily in the past 10 years during monitoring programs, impact assessments, or as part of the Strait of Georgia collaborative research project (Metro Vancouver/Fisheries and Oceans Canada/Natural Resources Canada – see [33] and references therein). Samples were either: (1) collected specifically to monitor background conditions and purposefully chosen for their remoteness to localized impacts; or (2) collected as remote reference samples during independent monitoring surveys for anthropogenic discharges (n = 1168 samples; see Fig. 1 for general sampling locations).

All grab samples collected have associated depth, sediment percent fines data (percent silt + clay particles <63 µm in diameter) and most have associated measurements (or extrapolated values – see below) of modified organic flux to sediments [31]. After exclusion of biological samples without reasonable flux data, 987 samples which included organic flux remained for data analyses. Organic flux measurements were obtained from 54 cores and 6 sediment-trap deployments throughout the Strait and surrounding fjords. Locations and data for all cores and traps are given in Burd et al. [34].

Analytical calculations for estimating organic carbon flux (sum of buried and oxidized organic material) from _210_Pb dated cores for the Strait of Georgia and surrounding fjords are described in Macdonald et al. [35], with justification for use of the method and comparison with other methods detailed in Johannessen and Macdonald [36]. The cores were all approximately 50 cm long. Immediately on recovery, the cores were sectioned for analysis into 1 cm intervals for the top 10 cm, 2 cm intervals for the next 10 cm, and 5 cm intervals for the remainder of the core. A sub-sample from each depth interval was analyzed by Flett Research Ltd., Winnipeg, Canada, for ^210^Pb and ^226^Ra to be used for radio-dating. The activity of supported ^210^Pb was determined as the average of the ^226^Ra activity measured at three depths (top, middle, and bottom) in each core, from the ingrowths of ^226^Rn over at least 4 days. Based on the assumption that bottom waters are always supplied with some oxygen (>2.5 mL L^−1^; [37]), there will be an active benthic community which mixes the surface sediments. Sedimentation and mixing rates in the sediment cores were determined using excess ^210^Pb profiles in sediments together with advective-diffusive models (see Johannessen et al. [38]), and assuming a constant supply of ^210^Pb and constant sedimentation rate. The depth of the surface mixed layer in each core was determined by visual measurement from the ^210^Pb profile. The incident flux of organic carbon (OC), the percent OC buried, and the percent OC oxidized, were estimated from the ^210^ Pb profiles of %OC measured in the sediment cores (see Johannessen et al. [39]). Although not an ideal measure of total sedimenting organic material (which is more accurately measured in bottom sediment traps), this is nevertheless a useful proxy for the amount of organic material that actually remains in sediments (taking resuspension into account) and is thus available for infaunal use.

A modified organic carbon flux measure was used in this study (described in [31]). This measure weights the organic carbon flux measured from cores by the δ^15^N ratio. This weighting assumes the lability of settling organic material is dependent on the age and amount of trophic reworking of that material. The higher the δ^15^N ratio, the less useable the organic material is for most organisms ([31] and references therein). Near-surface stable nitrogen isotopes (δ^15^N) were typically measured in the cores or in nearby surface grabs. Additional isotope data were available from extensive grab sample surveys in the southern Strait of Georgia (unpublished data from Environment Canada’s Ocean disposal program, [40]). The modification of organic flux was found to be necessary for understanding biological patterns in sediments with naturally high but mostly non-labile organic carbon content [31].

Organic carbon flux and δ^15^N values were assigned to nearby biological sample locations using an exponential variogram. The length scale of the variogram was fitted based on expected scale of variation in the geographic distribution of sedimentation rates, using a simple Kriging routine [41] programmed in MATLAB. Source data was filtered to replace clusters of very highly correlated points with their means to make the numerical solution more stable. This extrapolation was not possible for some sample locations, due to lack of nearby core data. Particular care was taken to avoid extrapolating in areas where cores were unavailable and unusual influences (such as river discharges) might affect localized flux patterns. Fortunately, cores were most numerous in the southeast portion of the main basin of the Strait, which is the area most affected by discharge from the Fraser River (the highest volume freshwater discharge in the Strait).

All biological samples were collected using 0.1 m^2^ Van Veen or Smith-MacIntyre grab samplers, screened on 1 mm sieves, initially preserved in 4–10% formalin and transferred to 70–95% ethanol for processing. All invertebrate taxa were identified to species, or to the lowest possible taxonomic level. In addition, all taxa were placed in a life stage category – ‘adults’, ‘subadults’, and ‘juveniles’. These groups may not be reflective of true life stages, given they are based strictly on size. However, we use the term ‘life stage’ to distinguish from size categorization used in this study (see below). All surveys followed strict quality control, and a detailed coding system has been used to maintain taxonomic consistency across studies [42].

Abundances were converted to estimates of wet-weight biomass for each taxon, in each sample. Biomass conversions were based on species-specific mean wet weight estimated for ‘adults, ‘subadults’ and ‘juveniles’ for each original survey area. Wet weights were estimated using preserved specimens only, thus any biomass shrinkage related to this preservation method [43], [44] should be consistent, and we examine only relative biomass proportions. Mean weights were applied consistently to convert abundance data to biomass data within each survey area, thus retaining information on geographic variation in body size within taxa. Very large organisms (e.g., >2 g) were weighed independently for each sample.

Wet weight was converted to carbon content using taxon-specific conversions [45], [34]. This conversion to carbon content (i.e., ash-free dry weight) removes the contribution to biomass from hard parts of various taxa (e.g. shells in bivalves, endoskeletons of echinoderms). Conversions used in this study are available in supplementary information (Table S1). Carbon content was converted to energy units (J) using the universal conversion factor of 46000 J/g C [45]–[47]. These conversion steps, although potentially introducing some uncertainty, are necessary to compare samples differing substantially in community composition across the region.

For the purposes of assigning accurate size categories, the average organic body mass was calculated for each ‘life stage’ of each taxon (species or lowest taxonomic level, as above), in each sample. These taxon/stage combinations were assigned a size class based on the average organic biomass. Size classes are logarithmic (log_2_), a standard practice for studies of the size structure of macorobenthic communities since the pioneering studies of Schwinghamer [1], [2] and Warwick [4]. There were a total of 24 size classes, ranging from 2^−6^ J (0.015625 J) to 2^16^J (65536 J) (log_2_ size classes are −6 to 16 respectively). Organic biomass in each size class was summed for each sample. We may lose some resolution in taking the average biomass of each life stage, and each taxon, instead of assigning each individual organism a size class. However, the scale of this study necessitates such averaging and this procedure accounts for the large range in body sizes characteristics of many benthic macro-invertebrate taxa.

### Response of Size Structure to Depth, Sediment Percent Fines, and Modified Organic Flux

We test the null hypothesis that organic biomass size structure is the same across gradients in depth, sediment percent fines, and modified organic flux. For the purposes of simplicity in analysis and interpretation, all three habitat factors were condensed into categorical ranges (Table 1). Depth and substrate categories were assigned based on steep gradients evident in community structure (e.g., richness and abundance), light penetration and habitat types as described for the Strait of Georgia by Burd et al. [31], [48]. These categories were found to be informative in the study of the trophic structure of the same communities in this dataset [32]. The greatest change in habitat conditions typically occurs within the shallow subtidal zone (0–10 m), and within the photic zone (and zone of major wave influence) that extends to approximately 25 m [31]. Habitat conditions tend to stabilize considerably below this depth, resulting in high diversity and abundance of organisms from 25–100 m depth [48]. This depth range was split arbitrarily into two even depth categories because of the large sample size in this depth range. Below 100 m, abundance and diversity of organisms decline steeply, and below 200 m biodiversity is uniformly low (Table 1; [47]). As for substrate categories, their delineation was also informed from past studies. Coarse sediments (with 0–10% fines) are trophically dissimilar to finer sediments [32]. In addition, P/B ratios for all invertebrate fauna tend to be highest in coarse sediments due to the prevalence of small organisms [34]. Very fine sediments (e.g., 85–100% silt + clay) are cohesive, retain more organic material [49], and subsequently are less permeable to oxygen in the absence of bioturbation (e.g., [50]). Mud content, therefore, is a significant predictor of macrobenthic biomass and abundance [51], as well as trophic structure [32].

**Table 1:** Categories for habitat factors (depth, sediment percent fines and modified organic flux) and their species diversity.

Habitat Factor	Category	Range	N	Total Pooled Species Richness	Mean Species Richness/sample (±SD)
Depth (m)	1	0–10	75	451	30.2±12.8
	2	11–25	66	575	41.1±22.3
	3	26–50	134	832	65.5±21.4
	4	51–99	650	1555	70.0±21.4
	5	100–200	63	693	57.3±23.4
	6	>201	180	567	18.4±12.6
Sediment % Fines	1	0–10	58	709	47.8±28.9
	2	11–20	31	744	86.1±47.6
	3	21–30	102	976	70.9±26.5
	4	31–50	250	1198	65.5±31.2
	5	51–85	294	1055	58.8±25.6
	6	86–100	433	920	45.9±24.1
Modified Organic Flux	1	0.0–0.5	248	723	51.0±25.8
(gCm^−2^yr^−1^/δ^15^N)	2	0.5–0.75	90	938	60.1±33.2
	3	0.75–1.0	177	873	56.8±22.2
	4	1.0–1.5	212	845	61.5±22.4
	5	1.5–3.0	136	974	80.3±36.6
	6	3.0–5.0	64	340	67.0±9.4
	7	5.0–10.0	64	345	63.1±12.5
	8	>10.0	70	317	74.3±9.0

Modified organic flux categories were constructed *a priori* using an arbitrary scale to ensure a reasonable number of samples in each category. The largest sample size category was in the lowest flux range (category 1), with samples well represented throughout the Strait, many of which may be food limited and show signs of biotic stress [31], [48]. Categories 2–5 had fluxes related to a steep gradient in invertebrate biomass and production in the Strait [34]. Samples in the three highest flux categories (6–8) have been found to exhibit very little change in somatic production of invertebrates [34], and included stations from various parts of the southern main basin of the Strait, which is influenced by high organic and inorganic discharge from the Fraser River (Fig. 1 and see [31], [48]).

Challenges exist in the comparison of many different faunal communities and habitat types. Over the entire Strait of Georgia, there is a broad range of total macrobenthic biomass [31], [48]. In addition, the biomass of macrobenthos is known to decline with depth [31], [34]. Therefore, it is most appropriate to standardize the biomass data in each sample by the total biomass. Thus the following community analyses were based on the distribution of proportional organic biomass among size categories in each sample.

Cluster analyses were used to examine the similarity of size structure among the habitat categories described above. For ease of interpretation, analyses were first performed separately for each habitat factor (depth, sediment percent fines, and modified organic flux). Each pair of categories was compared using Bray-Curtis dissimilarities [52]. From the resulting matrix of pair-wise dissimilarities, clusters were grouped using an agglomerative, hierarchical sorting procedure (unweighted pair group mean average sort) [53]. Using the replicate sample data for each category, a statistical bootstrap method called SIGTREE [54] was used to generate multiple simulations to test the generalized null hypotheses that there is no difference in size structure at each cluster linkage. The method is non-parametric, and makes no assumptions about the underlying distribution of the multivariate data. Rather, it examines the relative variability within and between habitat categories independently for each linkage, to determine whether or not a cluster grouping is statistically valid at a pre-determined probability level (α) of 0.01, resulting in a cumulative potential type I error for all linkages of α = 0.06 (6 linkages) for depth and sediment percent fines, and α = 0.08 (8 linkages) for modified organic flux.

To assess how biomass is distributed among size classes, we examined cumulative biomass size spectra for all environmental factor categories. These spectra are the proportion of total organic biomass in pooled samples (across each habitat category or y-axis) in each log_2_ size category (x-axis) (e.g, [2], [3], [6], [7], [9], [28], [55]. A size spectrum was constructed for each habitat factor separately (for each category) and also for combined factor categories. Pooled and mean species richness contributing to the cumulative biomass size spectra for each habitat category are found in Table 1.

Because of the potential inter-dependence of depth, percent fines, and modified organic flux, we examined the correlations between these habitat factors (Lin’s test of concordance –[56]). We also examined the response of size (organic biomass) structure to all three habitat factors together. We combined habitat factor analyses in two ways; (1) cluster analysis with categories including all three habitat variables, in which each habitat factor was re-categorized into two groups based on the most extreme dissimilarities from the single habitat factor cluster analyses described above; and (2) non-parametric multivariate methods in PRIMER-E v6 [57]. The RELATE procedure [57], [58] is used to test the null hypothesis of no agreement in multivariate pattern between two matching similarity matrices, a “biotic” one derived from the size structure (based on Bray-Curtis similarity), and an “abiotic” one derived from the environmental variables (Euclidean distance matrix, based on the continuous, non-categorized normalized environmental variables). A rank correlation coefficient (Spearman’s ρ) is used to determine how closely the rank order of similarities from the biotic matrix matches the rank order of similarities from the abiotic similarity matrix. The null hypothesis is that the rank order of similarities in biotic and abiotic matrices are not related. If the rank correlation coefficient (ρ) is significantly higher than 0 (p<0.01) based on 999 random permutations of samples that determined the value of ρ based on the null distribution, then the null hypothesis can be rejected.

If RELATE shows the biotic and abiotic matrices to be significantly related, the BEST procedure can infer which combination of environmental variables best explains the observed biotic matrix [59]. BEST explores the relationship between the biotic matrix and variables in the abiotic matrix by searching for the highest rank correlations between these matrices, when the abiotic matrix is based on different subsets of the environmental factors [57]. As with the RELATE procedure, all environmental variables were normalized prior to calculating the abiotic similarity matrix, and Spearman’s ρ rank correlation coefficient was used to compare matrices. We use the BIO-ENV option method in BEST, which examines all possible combinations of the biotic and abiotic matrices. The sensitivity of results for RELATE and BEST to a series of data transformations of both size (square root, log (x+1), presence/absence) and habitat data (log (X+1)) was assessed.

Since RELATE and BEST analyses are based on assessing similarities from the continuous sample data, these tests also serve the purpose of providing a comparison with the cluster patterns inferred from categorized habitat data. However, because of size limitations in the program, analyses were not possible in PRIMER-E without condensing the sample size. This will result in a loss of variability within the data. To do the analyses, we condensed the data from the original sample size down to 218 location averages (combining replicates over time), instead of using the 1168 original sample replicates in SIGTREE analyses described above. All data used in the PRIMER analyses were continuous, non-categorized data.

## Results

### Response of Size Structure to Depth, Sediment Percent Fines, and Modified Organic Flux

#### Depth

**Figure 2:**
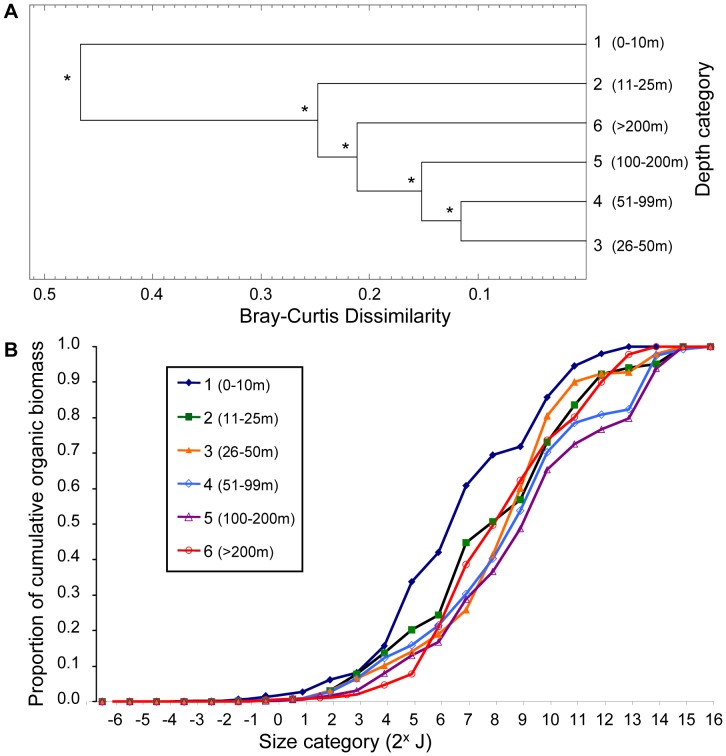
Response of size structure to water depth (m) categories. (**A**) Cluster analyses show relationships among depth categories based on their community size structure. SIGTREE analyses assess which relationships are statistically significant (asterisks indicate rejection of the null hypothesis (at α = 0.01) that the two groups being linked are homogeneous**.** (**B**) Cumulative biomass size spectra for each depth category show how biomass accumulates across size categories of macrobenthic organisms (based on log_2_ organic biomass).

Cluster and SIGTREE analyses for depth categories reveal significantly distinct size structure for all depth categories (Fig. 2A; p<0.00001). The maximum dissimilarity (0.48) occurred between category 1 (0–10 m) and the cluster of remaining depth categories (Fig. 2A). The cluster that contained depth categories 2–6 had a maximum within-cluster dissimilarity of 0.25 (Fig. 2A). The cumulative biomass size spectrum showed depth category 1 to be distinct because organic biomass accumulates in smaller size classes (2^4^ to 2^9^ J; Fig. 2B). The remaining depth categories tended to accumulate a higher proportion of organic biomass in larger size categories (>2^9^J). This was especially evident in depth categories 4 (51–99 m) and 5 (100–200 m), which had the highest proportion of biomass in very large organisms (>2^13^J; Fig. 2B). The largest infaunal organisms (2^14–^2^16^J) were *Molpadia intermedia* (Holothuroidea), *Brisaster latifrons* (Echinoidea), *Travisia pupa* (Polychaetea Sedentaria), *Cerebratulus californiensis* (Nemertea), *Glycera pacifica* and less commonly *Glycera robusta* (Polychaeta Errantia). These biomass size distributions were based on communities of varying species richness (Table 1), which apparently peaks at mid-depths (26–100 m) (Table 1).

#### Sediment percent fines

**Figure 3:**
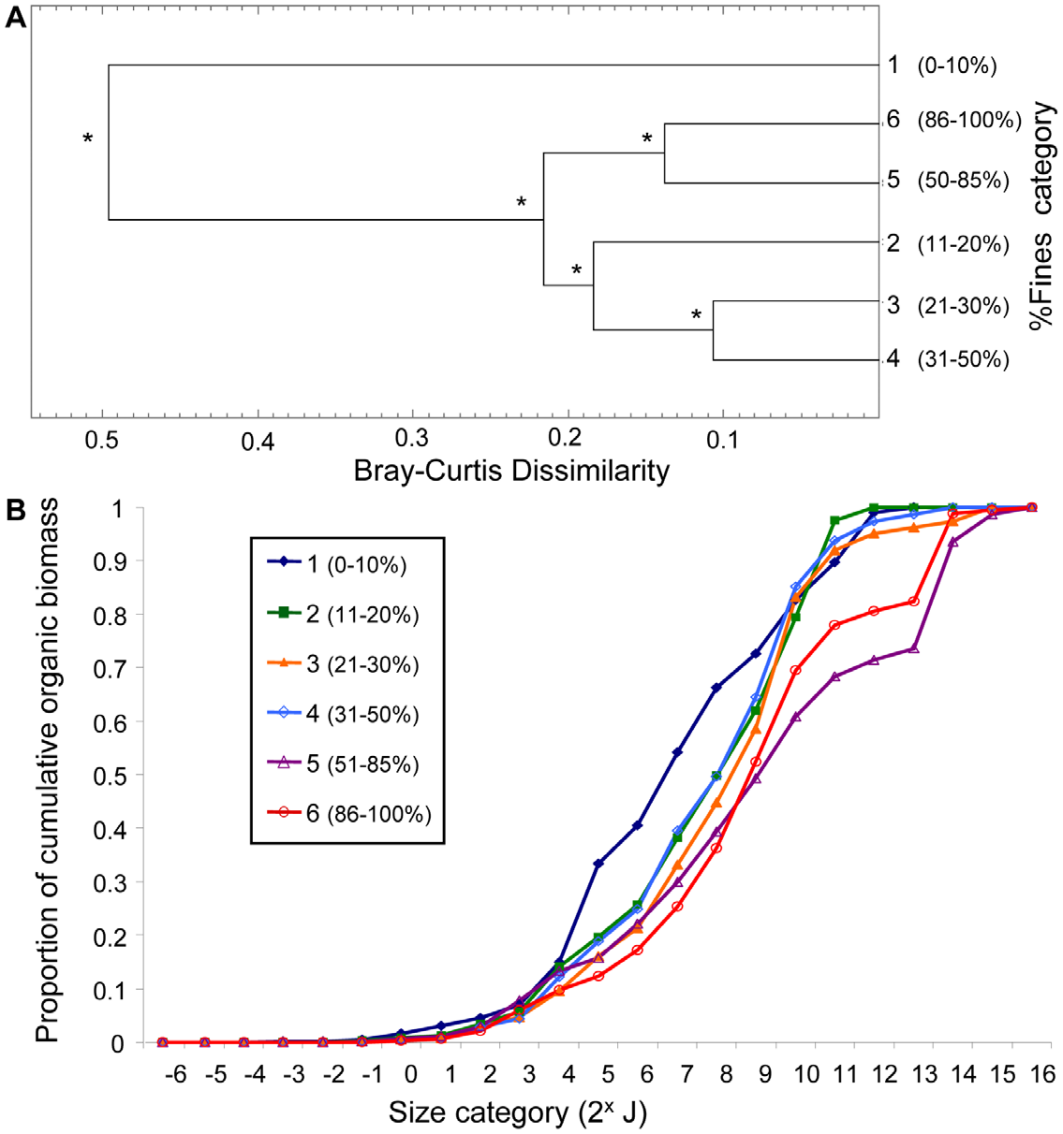
Response of size structure to sediment percent fines categories. (**A**) Cluster analyses show relationships among sediment percent fines categories based on their community size structure. SIGTREE analyses assess which relationships are statistically significant (asterisks indicate rejection of the null hypothesis (at α = 0.01) that the two groups being linked are homogeneous**.** (**B**) Cumulative biomass size spectra for each percent fines category show how biomass accumulates across size categories of macrobenthic organisms (based on log_2_ organic biomass).

Significant differences in size structure were evident between all sediment percent fines categories (Fig. 3A; p<0.00001). The SIGTREE analysis resulted in two maximally dissimilar groups: sediment category 1 (0–10% fines) and the remainder (categories 2–6; 11–100% fines). The dissimilarity between these two groups was 0.50. The cluster containing sediment categories 2–6 had a maximum within-group dissimilarity of 0.22. The cluster pattern showed a gradient response of size structure to sediment percent fines. The cumulative biomass size spectrum (Fig. 3B) showed size structure in the coarsest category 1 sediments (0–10% fine sediments) was distinct because biomass is predominantly in small organisms (2^4^ to 2^9^ J; Fig. 3B). The coarsest sediments had relatively low species richness (Table 1). Size spectra also showed that biomass accumulated progressively in larger organisms (>2^9^J) as the percentage of sediment fines increased, corroborating SIGTREE results (Fig. 3A). Therefore, the largest species were found in the finest sediments (Fig. 3B), which also had low species richness (Table 1).

#### Modified organic flux

**Figure 4:**
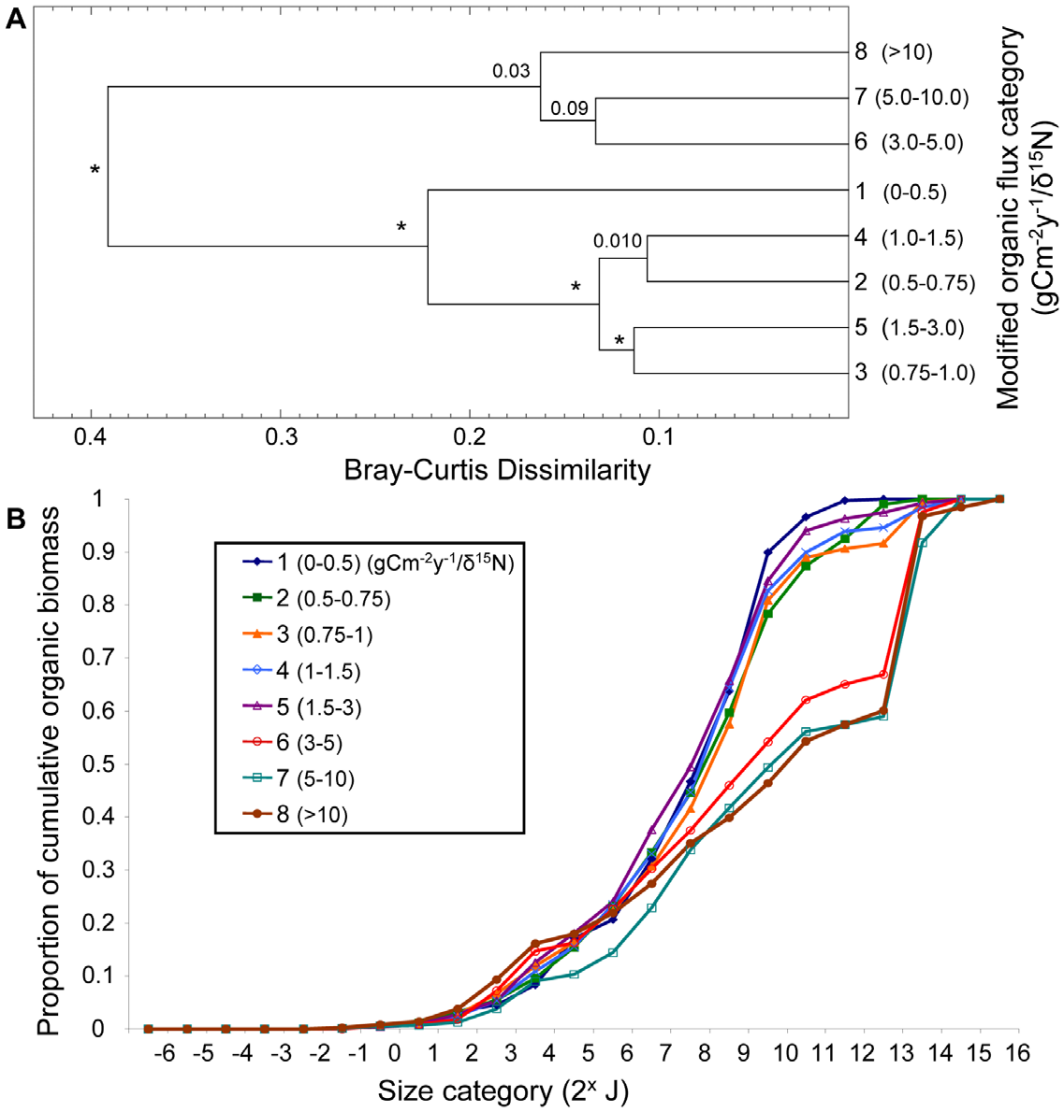
Response of size structure to modified (by quality, δ^15^N) organic flux categories (gC/m^2^/yr/δ^15^N). (**A**) Cluster analyses show relationships among modified organic flux categories based on their community size structure. SIGTREE analyses assess which relationships are statistically significant. Asterisks indicate p<0.0001, (and thus rejection of the null hypothesis at α = 0.01 that the two groups being linked are homogeneous). P-values >0.01 are indicated above nodes. (**B**) Cumulative biomass size spectra for each modified organic flux category show how biomass accumulates across size categories of macrobenthic organisms (based on log_2_ organic biomass).

Cluster and SIGTREE analyses revealed two distinct communities based on size structure: those occurring in samples experiencing a modified organic flux <3.0 gC/m^2^/yr/δ^15^N (flux categories 1–5), and those with fluxes greater than this threshold (flux categories 6–8). These two major clusters had a dissimilarity of 0.39 (Fig. 4A). Within the cluster of samples with modified flux <3 gC/m^2^/yr/δ^15^N, all categories were significantly distinct at p<0.01. Samples in the high flux categories 6–8 (>3.0 g C/m^2^/yr/δ^15^N) formed a significantly homogenous group (Fig. 4A). It is in these high flux categories that the largest organisms –most commonly *Molpadia intermedia, Brisaster latifrons,* and *Travisa pupa* - were found. Although these organisms are distributed widely in the Strait of Georgia, we found they attained their largest sizes and highest densities in the region exposed to the highest organic and inorganic flux (in the region of the Fraser River discharge – see Fig. 1). The cumulative biomass size spectra highlighted the striking difference in size structure between the cluster of modified organic flux categories 1–5 and that of categories 6–8 (at the 3 g C/m^2^/yr/δ^15^N threshold) (Fig. 4B). Samples in the high organic flux categories 6–8 (>3 gC/m^2^/yr/δ^15^N) clearly contained the majority of biomass in large organisms (>2^12^ J), whereas samples in categories 1–5 (0–3.0 gC/m^2^/yr/δ^15^N) showed a lack of the largest size classes, particularly in category 1 samples. Species richness among these categories did not differ greatly, but was relatively low in category 1 (Table 1).

#### Combined habitat factors

The habitat factors used in this study were poorly correlated (Lin’s concordance r values <0.19 among all variable pairs, p>0.05; Table 2), reflecting the hydrographic complexity of the Strait of Georgia. Thus these factors may not act co-dependently, but may instead have conflicting effects on size structure.

**Table 2:** Lin’s concordance test between habitat variables.

	Modified organic carbon flux	Sediment %fines
**Sediment** **%fines**	0.022	–
**Depth**	.0011	0.188

No correlations are significant (p>0.05 in all cases).

**Figure 5:**
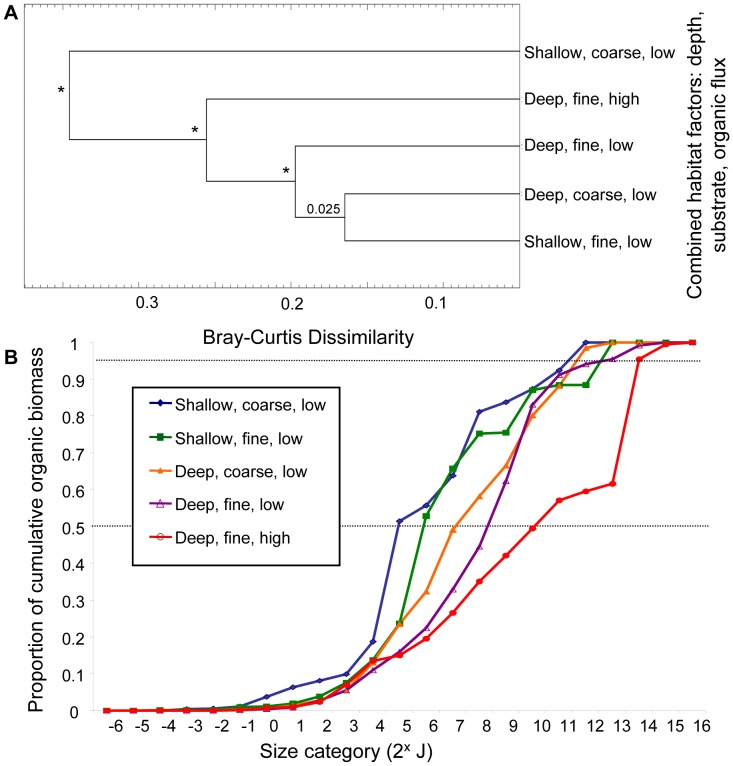
Response of size structure to combined habitat factors. Samples were re-categorized based on previous cluster analyses into shallow (<10 m) and deep (≥10 m), Coarse sediments (<10% fines) and Fine sediments (≥10% fines), and low organic flux (<3 gC/m^2^/yr/δ^15^N) and high organic flux (≥3 gC/m^2^/yr/δ^15^N) and analyzed together. (**A**) Cluster analyses show relationships among habitat categories based on their community size structure. SIGTREE analyses assess which relationships are statistically significant. Asterisks indicate p<0.0001, (and thus rejection of the null hypothesis at α = 0.01 that the two groups being linked are homogeneous). P-values >0.01 are indicated above nodes. (**B**) Cumulative biomass size spectra for each habitat category show how proportional biomass accumulates across size categories of macrobenthic organisms (based on log_2_ organic biomass). Horizontal lines are placed at 95% of total biomass and 50% of total pooled biomass.

Based on the above cluster analyses for individual habitat factors, samples were re-categorized into groups that reflect the maximal divergence in faunal size structure. For instance, the most distinct divergence in size structure in the cluster analysis and cumulative biomass size spectrum for depth was at 10 m, resulting in two new categories of <10 m and ≥10 m. Similarly, samples were grouped into <10% fines and ≥10% fines, and <3 gC/m^2^/yr/δ^15^N and ≥3 gC/m^2^/yr/δ^15^N. For convenience these categories can be distinguished by the terms deep/shallow, coarse/fine, and low/high flux respectively (reflecting the extremes that these categories represent). Five new combined habitat categories resulted, as not all possible combinations of habitat factors were present (Table 3). Using SIGTREE, the two most dissimilar and significantly distinct (p<0.01) community size structure groupings that emerged (Fig. 5A) were; (1) shallow, coarse, low flux samples, and (2) deep, fine, high flux samples. Both groups had significantly heterogeneous community size structure (p<0.0001). The deep/fine/low flux category (p<0.0001; Fig. 5A) also had significantly distinct size structure. The remaining two cluster groups (deep/coarse/low flux and shallow/fine/low flux) were not statistically distinguishable from each other (p = 0.025), but by extrapolation, were collectively distinct from all other cluster groupings.

**Table 3:** Recategorized combined habitat factor categories based on divergences in community size structure in cluster analyses.

Category	Habitat Factor	Range	N	Total Pooled Species Richness	Mean Species Richness/Sample (±SD)
Shallow/Coarse/Low flux	Depth	<10	32	264	33.1±14.3
	Sediment % fines	<10			
	Mod. organic flux	<3			
Shallow/Fine/Low flux	Depth	<10	43	354	28.1±11.3
	Sediment % fines	>10			
	Mod. organic flux	<3			
Deep/Coarse/Low flux	Depth	>10	26	581	66.0±32.1
	Sediment % fines	>10			
	Mod. organic flux	<3			
Deep/Fine/Low flux	Depth	>10	871	1866	57.0±31.7
	Sediment % fines	>10			
	Mod. organic flux	<3			
Deep/Fine/High flux	Depth	>10	196	669	62.8±18.4
	Sediment % fines	>10			
	Mod. organic flux	>3			

The cumulative biomass size spectra for these 5 categories supported the results of the SIGTREE analyses. The shallow/coarse/low flux and the deep/fine/high flux assemblages had the most extreme size distributions, containing higher proportions of total biomass in smaller and larger animals respectively. For instance, about 50% of total biomass in the shallow/coarse/low flux samples was found in organisms <2^4^J, whereas in the deep/fine/high flux samples 50% of the total cumulative biomass was found in organisms of >2^9^J (Fig. 5B). Notably, in the deep/fine/low flux sediments, the largest organisms (2^15^–2^16^J) as well as the smallest organisms (2^−6^ to 2^−5^ J) were missing. However, a large proportion of biomass was in moderately large organisms (95% of biomass in organisms <2^12^J) (Fig. 5B).

We further examined the combined effects of habitat factors on size structure using PRIMER-E. Various data transformations (square root, log (x+1), presence/absence) for both size and habitat data had no noticeable influence on the results or their interpretation, thus the results shown are those for the original log_2_ proportional organic biomass (biotic) size classes and normalized habitat factors (abiotic) data. The RELATE procedure confirmed that the size class (biotic) similarity matrix and the abiotic similarity matrix (depth, sediment percent fines, modified organic flux) had similar multivariate patterns. Thus the null hypothesis that these matrices were unrelated was rejected (p<0.01). However, the degree of correlation between the matrices was modest (Spearman’s rank correlation ρ = 0.382). The BEST routine [57] showed that depth alone resulted in the highest rank correlation with the biotic similarity matrix (BEST**,** Spearman’s ρ = 0.0.375, Table 4). All other individual factors had considerably lower correlations with the biotic matrix (Table 4). Modified organic flux alone resulted in the lowest rank correlation with the biotic similarity matrix (ρ = 0.157), which did not reflect the striking threshold response of size structure to high and low flux evident in the SIGTREE analyses and cumulative biomass size spectra (Figs. 4 and 5). Depth and sediment percent fines had a more gradient-type influence on size structure (Figs. 2 and 3 respectively), which was more readily recognized by the correlational PRIMER procedures. Combining habitat factors resulted in correlation values less than for depth alone. However, it cannot be concluded from this analysis that depth was the most important factor affecting size distributions. Rather, of the three habitat factors, depth produced the most linear response in size distributions. We conclude from all analyses in this study that all three habitat factors play a role in determining size structure of macrobenthos in the Strait of Georgia.

**Table 4:** Results of BEST (BIO-ENV) routine (PRIMER-E, PlymouthMarine Laboratory) based on Spearman’s correlation between size structure resemblance matrix (Bray-Curtis similarity, standardized organic biomass data)and normalized habitat matrix (Euclidean distance).

No. Variables	Habitat factors	Rho
1	depth	0.375
2	depth, organic flux	0.342
3	depth, fines,organic flux	0.338
2	depth, fines	0.325
2	fines, organic flux	0.277
1	fines	0.234
1	organic flux	0.157

Data are otherwise not transformed.

## Discussion

In this paper, we describe biomass distributions among size classes across a geographically extensive coastal sea [60], in order to establish a baseline for understanding how anthropogenic discharges and regional climate change may modify this functional aspect of marine sediment communities. The complexity of inputs, topography, and hydrographic conditions in the Strait of Georgia resulted in low correlations between depth, substrate type (sediment % fines), and the flux and quality of organic material (Table 2). This necessitated the examination of how each of these habitat factors (separately and in combination) affected natural patterns of biomass spectra throughout the Strait. By using an extensive regional database of macrobenthic samples, for which individual macrobenthic taxa and age classes from each sample were placed into ‘life stage’ (size) groups and subsequently into a spectrum of biomass categories, we have achieved adequate resolution to conclude that size structure shifts significantly between extremes of organic flux and quality, depth and substrate type found throughout the Strait.

One of the most striking patterns revealed herein is the distinct size distribution of macrobenthic communities from the southern main basin of the Strait influenced by high organic inputs from the Fraser River. At modified flux rates greater than 3 gC/m^2^/yr/δ^15^N (and up to 18 gC/m^2^/yr/δ^15^N in this region), size structure is remarkably homogeneous (Fig. 4). The samples from this area cover a considerable range of depths and a moderate range of sediment types. They also encompass a broad range in sedimentation regimes, suggesting the rate of organic input is the consistent driving factor affecting size structure in this part of the Strait. Both cluster analyses and cumulative biomass size spectra reveal the distinct size structure of this community.

The largest macro-infaunal organisms in the Strait of Georgia (including the largest representatives of the echinoderms *Molpadia intermedia* (Holothuroidea) and *Brisaster latifrons* (Echinoidea, Spantagoida)) are found in this region of high organic flux. These large echinoderms are closely related to other sedentary burrow-dwelling species well-known to play key roles in the ecosystem functioning of soft-bottom temperate habitats [11], [61], [62]. Such large burrowers can affect recruitment, growth and survival of a variety of organisms, and thus influence community biodiversity. The profound effects of their burrowing and feeding activities can include the delivery of food and solutes (e.g., oxygen) to subsurface sediments, alteration of sediment geo-chemical and physical makeup [14], and increased potential for grazing and subduction of smaller organisms [63] (and references therein).

These large, biomass-dominant burrow-dwellers are likely to have slow metabolisms and longer lifespans relative to smaller macrobenthic invertebrates. Communities dominated in biomass by large organisms are found to have low productivity relative to their standing biomass (see [34]). The predominance of these very large, slow growing organisms may explain why total invertebrate production in the southeastern Strait under the influence of the Fraser River tends to be unresponsive to the rate of organic flux [34]. We speculate the large burrow-dwelling echinoderms in this area may be reaching their maximal body sizes and densities, so that somatic production reaches a threshold, and will not change in response to increasing food input.

In contrast to the above described communities under the influence of the Fraser River, the significantly unique size structure of fauna from shallow (<10 m), coarse sediments (<10%fines), with low organic flux, had a much larger proportion of macrofaunal biomass held in smaller organisms (Fig. 5B). In general, rates of production relative to biomass have been found to be high in these areas [34]. Smaller organisms may account for more of the total proportional biomass in these areas because these coarse sediments have larger interstitial spaces that facilitate smaller, mobile forms [64]. Alternatively, this unique size structure may suggest that at >10% sediment fines, smaller forms become less competitive, perhaps due to increased predation by subsurface deposit feeders, which tend to be common in muddier sediments [32].

Between the two extreme environmental types described above (shallow/coarse/low flux and deep/fine/high flux), the macrofaunal communities in the Strait of Georgia appear to show a predominantly graduated response of size structure to both depth and substrate type. Duplisea & Drgas [28] and Duplisea [55] found that marine sediment particle size did not clearly influence benthic size structure; however, this larger-scale study (in terms of sample size as well as wide geographic region) shows that a subtle shift towards larger organisms occurs with increasing sediment percent fines. We speculate this distribution could result from the exclusion of large, sedentary burrowers from sandier, mobile substrates; possibly due to limitations on their ability to maintain an optimal burrow position in shifting sediments [65]. In addition, sandier sediments may not contain adequate nutrition for the large deposit feeders described above, as organic content increases with sediment % fines [49]. Regardless of the explanation for this pattern, the increasing size of organisms with increasing sediment % fines may explain the higher degree of bioturbation in fine sediments than in coarse sediments observed by Dashtgard et al. [66].

Despite a lack of strong correlation between depth and sediment % fines throughout the Strait, it remains difficult to separate their effects on community structure, because there is a depth threshold below which the coarsest sediments are virtually absent (see [32]). The biomass of large, biomass-dominant burrow-dwellers (which are primarily subsurface deposit feeders) clearly responds to both of these physical characteristics of habitat, driving patterns in trophic structure [32] and size structure (this study). However, it remains unclear why these organisms are found exclusively below the photic zone (e.g. >80 m for *Brisaster latifrons*, >25 m for *Molpadia intermedia*) when finer sediments also exist above these depths. More detailed analyses of the substrate may be required to reveal differences in its physical character that may be related to the distributions of these important taxa (e.g., clay mineralogy may reveal differences in sediment cohesiveness, organic content and stability [49], [51]).

There may also exist biological limits on the depth distributions of large burrow-dwellers, restricting them to deeper (>25 m) locations. These could include competitive interactions with other infaunal taxa, or potentially increased predation in these shallower areas; coupled with the possibility large burrowers are more able to thrive in low oxygen conditions or are less easily buried in regions with high sedimentation. These traits could be be mediated by body size. Additionally, large, long-lived organisms may be more able to thrive in depths with temporally and spatially patchy food input, due to their presumably lower metabolic rates. These are clearly speculative explanations for the observed patterns, and bear further investigation considering the relative abundance of these large burrowers, and their potential importance for the function of these ecosystems.

A contributing factor to the shift in biomass towards larger organisms in deep, fine sediments is the gradual loss of smaller macrofaunal taxa as both sediment percent fines and depth increase. In deep, fine sediments with low flux conditions, a third significantly distinct size assemblage was evident (Fig. 5A): one that lacks very large and very small fauna. Some of the large burrowers common in the high flux areas of the Strait were present in this habitat type, although they were consistently smaller than individuals found in the high flux regions. Their small body sizes could result from limited nutrition in these areas. Overall, the shift in faunal size structure from shallow, coarse sediments to deep, fine sediments reflects similar patterns found in trophic structure for the region [32].

BEST and RELATE analyses (PRIMER-E) provide an independent test of the patterns evident from cluster and SIGTREE analyses, except that they use the original continuous, non-categorized habitat factors to investigate the relationship between biotic and abiotic similarity matrices. These results are not clearly compelling (possibly due to a smaller sample size), but do illustrate that all three habitat factors influence community size structure in different ways. Thus, combined analyses actually decrease the agreement between the biotic and abiotic similarity matrices. This may be explained by the low correlation between the habitat factors, but is more likely due to the threshold type of response of size structure to extremes of all three habitat factors, combined with gradient responses to less extreme conditions.

A clear understanding of the biomass distribution among size classes across this coastal sea establishes baselines for future studies of natural phenomena as well as anthropogenic discharges and climate change. In particular, impacts influencing the abundance and distribution of large burrow-dwellers may have consequences for overall diversity and production of macrobenthic communities in coastal seas. Physical factors not examined in this study which may be important mediators of size distribution patterns in Strait of Georgia benthos include near-bottom and sediment oxygenation [21], particulate sedimentation, and other disturbances [67]. Investigation of these factors may help in understanding the reasons behind the patterns investigated in this paper, shedding light on some of the speculation above.

In conclusion, size structure of soft-bottom macrobenthic communities is clearly a complex response to the three habitat variables investigated here. Macrobenthic size structure tends to respond to water depth and sediment percent fines in a mostly graduated manner, with shallow, coarse sediments containing a proportionally high amount of organic biomass in small organisms, and deep, fine sediments containing a relatively high proportion of biomass in larger organisms. Communities between these two extremes tend to exhibit intermediate size structure. Of these two factors, size distributions respond to depth in the most linear way. Size distributions respond in a more punctuated way to organic flux and quality. When all three habitat factors were considered concurrently, several significantly distinct size distributions were evident, including;

1. A broad size spectrum assemblage with a highest proportion of biomass in the largest organisms found in the Strait, characteristic of sediments under the influence of the Fraser River discharge;
2. A predominantly small organism assemblage which lacks the largest size classes, typical of coarse (<10% fines), shallow (<10 m) and low flux sediments and;
3. A deep, fine sediment and low flux assemblage tending to lack both the largest and smallest size classes.

The gradient of size structures found for other combinations of the three habitat factors examined is narrow, with relatively low dissimilarity. This consistency is striking, considering the large geographic scale and associated hydrographic and biological diversity encompassed by this study.

## Supporting Information

Table S1 **Conversion factors (percent) used for g wet weight to g organic carbon, based on literature sources.** Where quoted values were similar, multiple authors are cited, and may be based on the same sources. Values quoted for specific taxa or sub-groups were used where applicable. Because P/B ratios were calculated using the formula of Brey (2001) [1], quoted values used in that handbook were used where applicable.
(DOCX)
