# Supplementary material for: Size Structure of Marine Soft-Bottom Macrobenthic Communities across Natural Habitat Gradients: Implications for Productivity and Ecosystem Function
Source: PLoS One. 2012 Jul 20;7(7):e40071. doi: 10.1371/journal.pone.0040071 (PMC3401224; doi:10.1371/journal.pone.0040071)
Supplement: Table S1 — Conversion factors (percent) used for g wet weight to g organic carbon, based on literature sources. Where quoted values were similar, multiple authors are cited, and may be based on the same sources. Values quoted for specific taxa or sub-groups were used where applicable. Because P/B ratios were calculated using the formula of Brey (2001) [1], quoted values used in that handbook were used where applicable. (DOCX) [file pone.0040071.s001.docx]

Supporting Table S1. Literature conversion values and sources for wet weight to % organic carbon .The conversion from g organic carbon to energy units, as required in the production model used was 46 kj/g organic carbon [3],[2],[8].

| **General faunal groups** | **% organic carbon (from wet weight)** | **Reference** |
| --- | --- | --- |
| Actinaria | 6.3 | [1] |
| Anthozoa (general) | 6.9 | [2] |
| Octocorals | 5.87 | [3] |
| Anemones | 7.46 | [3] |
| *Cerianthiopsis americanus* | 5.24 | [4] |
| Brachiopoda | 5.43 | [3] |
| Bryozoa | 3.65 | [5], [6], [2] |
| Bivalvia | 2.75 | [7], [8], [2] |
| *Clinocardium ciliatum* | 1.5 | [5],[6] |
| Opisthobranch - shelled | 6.9 | [5],[6] |
| Opisthobranch-non shelled | 8.58 | [5] |
| Prosobranchia | 3.4 | [9] |
| Crustacea (general) | 8 | [2] |
| Amphipoda | 4.5 | [9], [4] |
| *Ampelisca abdita* | 6.48 | [4] |
| *Corophium sp* | 2.03 | [4] |
| *Jassa pelagica* | 7.14 | [4] |
| *Leptocheirus pinguis* | 4.08 | [4] |
| Cirripedia | 1.95 | [2] |
| Cumacea | 3.75 | [2] |
| Decapoda | 9 | [2] |
| Isopoda | 7.1 | [5], [6], [2] |
| Leptostraca | 7.5 | [7] |
| Mysidacea | 7.75 | [5], [6] |
| Ostracoda | 6 | [10] |
| Tanaidacea | 2.9 | [9] |
| Miscellaneous crustacea | 8.45 | [2] |
| Hirudinea | 6.5 | [11] |
| Hydrozoa | 2.3 | [9] |
| **Echinodermata** |  |  |
| Asteroidea | 6.2 | [2] |
| Echinoidea | 2.45 | [2] |
| Holothuroidea | 5.6 | [2] |
| Ophiuroidea | 4.5 | [2] |
| Echiura | 5.1 | [9] |
| Entoprocta | 3.65 | [5], [6], [2] |
| Hemichordata | 3.8 | [12] |
| Kinorhyncha | 11.6 | [2] |
| Aplacophora | 5.7 | [9] |
| Nemertea | 10 | [5], [6], [2] |
| Phoronida | 5.1 | [9] |
| Platyhelminthes | 12.6 | [5], [6] |
| Oligochaeta | 16.15 | [5], [6] |
| Polychaetes (general) | 5.1 | [9] |
| Errantiate polychaetes | 8.5 | [5],[6], [8], [2] |
| *Nephtys incise* | 7.49 | [4] |
| Sedentariate polychaetes | 7.25 | [5], [6], [8], [2] |
| *Mediomastus ambiseta* | 4.76 | [4] |
| *Streblospio benedicti* | 4.8 | [4] |
| *Polydora ligni* | 7.53 | [4] |
| *Chaetozone sp.* | 10.76 | [4] |
| *Ampharetidae* | 6.54 | [4] |
| Polyplacophora | 13.6 | [5],[6] |
| Pycnogonida | 10.4 | [1] |
| Pogonophora | 5.1 | [9] |
| Porifera | 3.75 | [2] |
| Priapula | 3.25 | [5], [6], [2] |
| Scaphopoda | 4 | [9] |
| Sipuncula | 5.2 | [9], [2] |
| Urochordata | 1.5 | [1], [2] |
| **Permanent meiofauna** |  |  |
| Foraminifera | 2 | [10] |
| Harpacticoida | 8 | [10] |
| Nematoda | 9 | [10] |

1. Galeron, J, Sibuet, M, Mahaut, M-L, Dinet, A (2000) Variation in structure and biomass of the benthic communities at three contrasting sites in the tropical Northeast Atlantic. Mar Ecol Prog Ser 197, 121-137.

2. Brey, T (2001) Population dynamics in benthicinvertebrates. A virtual handbook. Alfred

Wegener Institute for Polar and Marine Research, Germany. <http://www.awi-bremerhaven.de/Benthic/Ecosystem/FoodWeb/Handbook/main.html>.

3. Steimle, F, Terranova, R (1985) Energy equivalents of amrine organisms from the continental shelf of the temperate northwest Atlantic. J Northw Atl Fish Sci 6: 117-124.

4. Frithsen, JB, Rudnick, DT, Doering, PH (1986) The determination of fresh organic carbon weight from formaldehyde preserved macrofaunal samples. Hydrobiologia 133, 203-208.

5. Ricciardi, A and Bourget, E (1998) Weight-to-weight conversion factors for marine benthic macroinvertebrates Mar Ecol Prog Ser 163, 245-251.

6. Clarke, A (2008) Ecological stoichiometry in six species of Antarctic marine benthos. Mar Ecol Prog Ser 369: 25-37.

7. Lie, U (1968) A quantitative study of benthic infauna in Puget Sound. Fiskeridirektoratet. Skrifter. Serie Havundersoekelser 14, 556pp.

8. Cauffope, G, Heymans, S (2005) Energy contents and conversion factors for sea lion’s prey. UBC Fisheries Centre Research Reports 13, 1.

9. Rowe, G (1983) Biomass and production in the deep-sea macrobenthos. In: Rowe, G. (Ed.), The sea. Deep-Sea Biology Vol 8. Wiley, New York. Pp. 97-121.

10. Rudnick, DT, Elmgren, R, Frithsen, J (1985) Meiofaunal prominence and benthic

seasonality in a coastal marine ecosystem. Oecologia 67, 157-168.

11. Leuven, RS, Brock, TC and van Druten, HA (1985) Effects of preservation on dry- and ash-free dry weight

biomass of some common aquatic macro-invertebrates. Hydrobiologia 127, 151-159.
